# Effect of liraglutide treatment on body mass index and weight parameters in children and adolescents with type 2 diabetes: Post hoc analysis of the ellipse trial

**DOI:** 10.1111/ijpo.12778

**Published:** 2021-02-25

**Authors:** Megan O. Bensignor, Eric M. Bomberg, Carolyn T. Bramante, T. V. S. Divyalasya, Paula M. Hale, Chethana K. Ramesh, Kyle D. Rudser, Aaron S. Kelly

**Affiliations:** ^1^ Department of Pediatrics University of Minnesota Medical School Minneapolis Minnesota USA; ^2^ Center for Pediatric Obesity Medicine University of Minnesota Medical School Minneapolis Minnesota USA; ^3^ Division of General Internal Medicine University of Minnesota Medical School Minneapolis Minnesota USA; ^4^ Novo Nordisk Bangalore India; ^5^ Novo Nordisk Inc. Plainsboro New Jersey USA; ^6^ Division of Biostatistics University of Minnesota School of Public Health Minneapolis Minnesota USA

**Keywords:** anti‐obesity agents, body mass index, liraglutide, paediatric obesity, weight

## Abstract

**Background:**

Weight loss in children and adolescents with type 2 diabetes (T2D) is associated with improved glycaemic control.

**Objectives:**

To assess the effects of liraglutide vs placebo on body mass index (BMI) and weight parameters in children and adolescents with T2D using data from the ellipse trial (NCT01541215).

**Methods:**

The ellipse trial randomized participants (10‐<17 years old, BMI >85th percentile, T2D, glycated haemoglobin [HbA_1c_] 7.0%‐11.0% [if diet‐ and exercise‐treated] or 6.5% to 11.0% [if treated with metformin, basal insulin or both]) to liraglutide or placebo. This *post‐hoc* analysis evaluated changes from baseline to weeks 26 and 52 in absolute BMI, percent change in BMI and other weight‐related parameters. Changes were assessed by liraglutide overall (all doses) and liraglutide by dose (0.6, 1.2 and 1.8 mg/day) vs placebo using a pattern mixture model of observed data, with missing observations imputed from each treatment group.

**Results:**

In total, 134 participants were included. There were statistically significant differences between groups in certain parameters, including absolute BMI (estimated treatment difference [ETD] –0.89 kg/m^2^; 95% confidence interval [CI] –1.71,–0.06) and percent change in BMI (ETD –2.73%; 95% CI –5.15,–0.30) at week 52, but none at week 26. Dose‐dependent effects were not observed for liraglutide vs placebo for all BMI/weight parameters.

**Conclusions:**

Compared with placebo, liraglutide was associated with statistically significant reductions in BMI/weight parameters at week 52, but not week 26, in children and adolescents with T2D.

## 1 INTRODUCTION

The incidence of type 2 diabetes (T2D) in children and adolescents in the United States increased annually by 7% between 2002 and 2012.^1^ T2D is often a complication of obesity, with the majority of children and adolescents having obesity at the time of T2D diagnosis.^2^ Compared with the development of T2D in adulthood, in children and adolescents, deterioration of β‐cell function and complications such as kidney disease, dyslipidaemia, retinopathy and hypertension are seen earlier in the disease course.^3^, ^4^ Despite ongoing evidence of disease progression and weight gain with lifestyle management and metformin therapy, metformin and insulin were the only US Food and Drug Administration (FDA)‐approved treatments for children and adolescents with T2D until 2019.^5^, ^6^, ^7^ Although weight loss can improve insulin resistance and glycaemic control, as well as delay or prevent the need to initiate insulin, metformin does not consistently elicit large weight reductions.^8^, ^9^, ^10^ Furthermore, treatment with insulin often leads to weight gain, which can be perceived by patients as a negative consequence of treatment and, therefore, acts as a barrier to adherence.^11^ This can lead to poorer glycaemic control and further increased risk of complications.^11^ Moreover, insulin‐associated weight gain can worsen insulin resistance, resulting in the escalation of insulin therapy and, therefore, promote additional weight gain.^11^

With the 2019 FDA approval of the daily injectable glucagon‐like peptide‐1 receptor agonist (GLP‐1 RA) liraglutide 1.8 mg/day (Victoza), there is now another effective treatment, with an acceptable safety profile, for T2D in children and adolescents aged ≥10 years.^12^ Liraglutide improves glycaemic control in children and adolescents with T2D and may lead to weight reduction, as seen in adults.^12^ Liraglutide has also been indicated at a higher dose of 3.0 mg/day (Saxenda) for chronic weight management in adults with obesity (body mass index [BMI] ≥30 kg/m^2^) or overweight (BMI ≥27 kg/m^2^) in the presence of at least one weight‐related condition.^13^ Although no GLP‐1 RA trials to our knowledge have been published in children and adolescents with T2D with a primary focus on weight reduction, GLP‐1 RAs have been shown to reduce body weight in adults with obesity, with or without T2D, and in children and adolescents with obesity but without diabetes, by 0.5‐5.0 kg.^13^, ^14^, ^15^, ^16^, ^17^, ^18^, ^19^ A randomized controlled trial demonstrated superiority of liraglutide 3.0 mg/day compared with placebo in reducing BMI SD score (a measure of relative weight, matched for age and sex) and other BMI‐ and weight‐related outcomes in children and adolescents aged 12 to <18 years with obesity (estimated treatment difference [ETD] in BMI SD score −0.22; 95% confidence interval [CI] −0.37, −0.08; *P* = .002).^20^

The Evaluation of Liraglutide in Pediatrics with Diabetes (ellipse) trial generated the data that resulted in FDA approval of liraglutide in children and adolescents with T2D.^21^ The ellipse trial was a double‐blinded, multinational trial, which randomized participants aged 10‐<17 years with T2D to liraglutide or placebo. In this trial, liraglutide doses up to 1.8 mg/day improved glycaemic control in children and adolescents with T2D and overweight or obesity compared with placebo.^21^ No significant difference in BMI z‐score (a confirmatory secondary endpoint) was observed with liraglutide vs placebo at week 26, but a significant difference was observed with liraglutide vs placebo at week 52 (ETD −0.18 (95% CI −0.33, −0.03).^21^ Furthermore, reductions in mean body weight were maintained only among those receiving liraglutide at week 52 (−1.91 kg with liraglutide vs +0.87 kg with placebo).^21^

A limitation of the BMI z‐score (also called BMI SD score) is that the sample size within age‐sex groupings from the Centres for Disease Control and Prevention (CDC) growth chart data set, from which the score is derived, is too sparse to estimate higher percentiles with adequate statistical reliability.^22^ As such, the BMI z‐score scale within the upper part of the distribution is foreshortened because of how the BMI distribution is skewed, resulting in the lower centiles being closer together than the upper centiles.^23^ Therefore, the BMI z‐score data set yields misleading results when applied to cohorts consisting of participants with severe obesity, as was the case in ellipse.^24^, ^25^, ^26^, ^27^

The objective of this post hoc, secondary analysis of the ellipse trial was to evaluate the effects of liraglutide, including its dose dependency, on BMI and weight parameters in children and adolescents with T2D and either overweight, obesity, or severe obesity. Some BMI and weight parameters are generally considered to be more appropriate to use in paediatric populations, as compared with the BMI z‐score,^23^ and so were included in this analysis. Due to the implications of insulin use on weight gain, an exploratory aim was to compare insulin rescue use between liraglutide and placebo groups.

## 2 METHODS

### 2.1 Design and participants of the ellipse trial

The ellipse trial (NCT01541215 https://clinicaltrials.gov/ct2/show/NCT01541215) was a phase 3a, randomized, double‐blinded, placebo‐controlled, multinational and multicentre trial investigating liraglutide in children and adolescents with T2D. The full study details have been reported previously.^21^ Briefly, the ellipse trial included 84 sites in 25 countries and enrolled participants over a period of 4 years and 4 months. Key inclusion criteria were age 10 to <17 years, BMI >85th percentile for age and sex, and glycated haemoglobin (HbA_1c_) 7.0% to 11.0% (53‐97 mmol/mol; if diet‐ and exercise‐treated) or 6.5% to 11.0% (48‐97 mmol/mol; if treated with metformin, basal insulin or both).

The ellipse trial protocol was approved by an independent ethics committee or institutional review board at each site (list of participating sites can be found in the Supplementary Material of the original study^21^). Written informed consent and assent were obtained from all parents/guardians and participants, respectively, as applicable.^21^ After screening, participants who were not already on a stable metformin dose underwent an 11‐ to 12‐week run‐in, during which metformin was titrated for 3 to 4 weeks to a maximum tolerated dose of up to 2000 mg/day, followed by maintenance for 8 weeks.^21^ For those who were treated with basal insulin at randomization, their insulin dose was reduced by 20%.^21^ Participants were then randomized 1:1 to subcutaneous liraglutide (1.8 mg/day or maximum tolerated dose) or placebo, both in combination with metformin (with or without basal insulin) for a 26‐week double‐blinded period, followed by a 26‐week open‐label extension.^21^ At week 26 (primary endpoint assessment), treatment allocation was unblinded. During the 26‐week open‐label extension, participants assigned to liraglutide continued their treatment unchanged, whereas those assigned to placebo discontinued the placebo injections, continued metformin (with or without basal insulin) treatment and were not started on liraglutide treatment.^21^

Participants in the liraglutide group initially received 0.6 mg/day, and were up‐titrated to 1.2 and 1.8 mg/day based on tolerability and efficacy of the dose (as determined based on whether the participant's average fasting plasma glucose [FPG] was >6.1 mmol/L [110 mg/dL]) on the three consecutive days preceding the dose‐escalation visit.^21^ The dose was up‐titrated at the investigator's discretion within a 3‐week period, after which a stable dose was maintained.^21^ After completion of liraglutide dose escalation, insulin dose could be increased within a 4‐week period, but not to more than the dose at randomization.

### 2.2 BMI and weight parameters

This *post‐hoc* analysis examined the following BMI and weight parameters:

- Absolute BMI (kg/m^2^)
- Percent change in BMI (%)
- Percentage of the 95th percentile for BMI (%BMIp95, %)
- Difference in BMI from 95th percentile BMI (ΔBMIp95, kg/m^2^)
- Percentage of median (50th percentile) BMI (%)
- Tri‐ponderal mass index (TMI, kg/m^3^)—the ratio of body weight to height cubed^28^
- Waist circumference (WC) (cm)

Each of these BMI and weight parameters were assessed for liraglutide overall and by liraglutide dose (0.6, 1.2 and 1.8 mg/day), all vs placebo. Participants were categorized according to the dose taken for the longest time during the maintenance period (double‐blind and open‐label periods combined). Weight was measured at site visits at screening, randomization and weeks 6, 10, 14, 20, 26, 30, 36, 42, 48 and 52; height and WC were measured at site visits at screening, randomization and weeks 14, 26 and 52. Weight and WC were measured with participants wearing light clothing, and weight and height were measured with participants not wearing shoes. The World Health Organization (WHO) growth curves were used to determine the BMI percentile.^29^, ^30^

### 2.3 Statistical analyses

Descriptive characteristics were summarized overall, with mean (SD) for continuous variables and frequency with percentage for categorical variables. The mean change from baseline to week 52 for each parameter was compared between placebo group and either liraglutide group overall or dose‐attained group. The primary analysis was a pattern mixture model (PMM) of observed data with missing observations imputed from each randomized treatment group based on multiple (×10,000) imputations. Missing data were imputed by selecting a random observation from a normal distribution centred at the value predicted by the regression model and with variance analogous to predicting a new observation in the regression analysis. We also performed a supporting analysis using a PMM of observed data with missing observations imputed from the placebo treatment group (as per the main analysis for the primary outcomes^21^). This supporting analysis was considered to be a more conservative approach than the primary PMM analysis used in this analysis. The data for week 26 and 52 were analysed with an ANCOVA model containing treatment (consisted of four groups: placebo and the three doses of liraglutide), sex and age group as fixed effects and baseline value as a covariate. ETDs and CIs were combined using Rubin's formula and CIs and *p* values were calculated using model‐based SE. Changes in the percentage of participants using insulin from baseline to week 52 were analysed using McNemar's test. The significance level was set at 5%. No adjustments were performed for multiple comparisons as these were *post hoc*, secondary analyses. Statistical Analysis Software version 9.4 (SAS Institute, Cary, North Carolina) was used to perform all of the analyses.

## 3 RESULTS

### 3.1 Participant demographics

In total, 134 participants received at least one dose of liraglutide (n = 66) or placebo (n = 68),^21^ and were therefore included in our analysis. All but eight participants (6%) had severe obesity (BMI ≥120% of the 95th percentile) at baseline (Table 1). Patient baseline demographics were generally well balanced between treatment groups. Within the liraglutide group, after the dose titrations as described in Section 2, there were 19 participants in the 0.6 mg group, 12 in the 1.2 mg group and 35 in the 1.8 mg group (Table S1, Supporting Information).

**TABLE 1:** Baseline demographics of participants in the ellipse trial

	Liraglutide (n = 66)	Placebo (n = 68)	Total (n = 134)
Age, years	14.6 (1.7)	14.6 (1.7)	14.6 (1.7)
Sex, n [%]			
Male	25 [37.9]	26 [38.2]	51 [38.1]
Race, n [%]			
White	42 [63.6]	45 [66.2]	87 [64.9]
Black or African American	9 [13.6]	7 [10.3]	16 [11.9]
Asian	10 [15.2]	8 [11.8]	18 [13.4]
American Indian or Alaska Native	2 [3.0]	1 [1.5]	3 [2.2]
Native Hawaiian or other Pacific Islander	0 [0.0]	0 [0.0]	0 [0.0]
Other	3 [4.5]	7 [10.3]	10 [7.5]
Tanner staging (females), n [%]^a^			
I	1 [2.4]	0 [0.0]	‐
II	2 [4.9]	0 [0.0]	‐
III	4 [9.8]	10 [23.8]	‐
IV	8 [19.5]	9 [21.4]	‐
V	26 [63.4]	23 [54.8]	‐
Tanner staging (males), n [%]^b^			
I	2 [8.0]	0 [0.0]	‐
II	1 [4.0]	3 [11.5]	‐
III	2 [8.0]	6 [23.1]	‐
IV	9 [36.0]	11 [42.3]	‐
V	11 [44.0]	6 [23.1]	‐
Duration of diabetes, years	1.9 (1.7)	1.9 (1.3)	1.9 (1.5)
BMI, kg/m^2^	34.6 (10.9)	33.3 (7.4)	33.9 (9.3)
%BMIp95	175.3 (55.4)	168.8 (37.4)	172.0 (47.1)
TMI, kg/m^3^	21.2 (7.1)	20.3 (4.7)	20.8 (6.0)
Waist circumference, cm	106.1 (20.7)	104.3 (15.0)	105.2 (18.0)
Severe obesity^c^, n [%]			
Yes	61 [92.4]	65 [95.6]	126 [94.0]
HbA_1c_, %	7.9 [1.4]	7.7 [1.3]	7.8 [1.3]

*Note:* Data are observed mean (SD) unless otherwise stated.

Abbreviations: %BMIp95, percentage of the 95th percentile for BMI; BMI, body mass index; HbA_1c_, glycated haemoglobin; n, number of participants; SD, standard deviation; TMI, tri‐ponderal mass index.

^a^ Based on available data for 41 and 42 patients receiving liraglutide and placebo, respectively.

^b^ Based on available data for 25 and 26 patients receiving liraglutide and placebo, respectively.

^c^ Severe obesity is defined as BMI ≥120% of 95th percentile.

### 3.2 Changes in BMI and weight parameters with liraglutide

**FIGURE 1:**
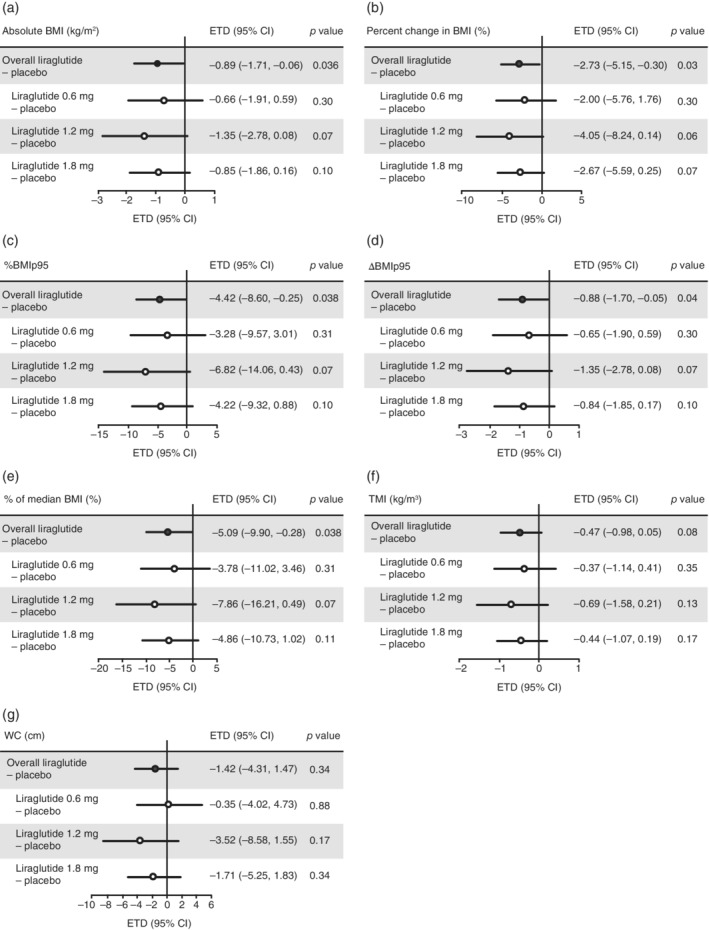
Differences in mean change from baseline in BMI and weight parameters between placebo and liraglutide overall and by liraglutide dose, at week 52 of the ellipse trial. Data analysed using a PMM of observed data with missing observations imputed within each randomized treatment group based on multiple (×10,000) imputations. Data were then analysed with ANCOVA model containing treatment (consisted of four groups: placebo and the three doses of liraglutide), sex and age group as fixed effects and baseline value as covariate. ETDs and CIs were combined using Rubin's formula. Participants categorized by dose taken for the longest time during the maintenance period, which consisted of the double‐blind and open‐label parts of the trial. %BMIp95, percentage of the 95th percentile for BMI; ΔBMIp95, difference in BMI from 95th percentile BMI; BMI, body mass index; CI, confidence interval; ETD, estimated treatment difference from baseline; PMM, pattern mixture model; TMI, tri‐ponderal mass index; WC, waist circumference

In the primary analysis, in which missing observations were imputed from each randomized treatment group, participants in the overall liraglutide group experienced significant differences in absolute BMI (ETD −0.89 kg/m^2^; *P* = .036), percent change in BMI (ETD –2.73%; *P* = .028), %BMIp95 (ETD –4.42%; *P* = .038), ΔBMIp95 (ETD –0.88 kg/m^2^; *P* = .037) and percentage of median BMI (ETD −5.09%; *P* = .038) vs placebo from baseline to week 52 (Figure 1A–E). Changes in TMI and WC from baseline to week 52 for the overall liraglutide group compared with the placebo group were not significantly different (*P >* .05 for both) (Figure 1F and G). There were no significant differences in liraglutide overall vs placebo for all BMI and weight parameters from baseline to week 26 (Table S1). No significant dose‐dependent effects were observed when comparing the three doses of liraglutide (0.6, 1.2 and 1.8 mg/day) with placebo from baseline to week 26 (Table S1) or week 52 (Figure 1).

The supporting analysis, which used the PMM with missing observations imputed from the placebo group, showed similar results to the primary analysis (Table S2). However, while no significant effect was observed with liraglutide 1.2 mg/day at week 52 in the primary analysis, the liraglutide 1.2 mg/day dose group differed significantly from placebo in the supporting analysis at week 52 for change in absolute BMI (ETD −1.41 kg/m^2^; *P =* .050), percent change in BMI (ETD –4.15%; *P =* .048), %BMIp95 change (ETD –7.15; *P* = .050), ΔBMIp95 change (ETD –1.42 kg/m^2^; *P* = .049) and change in percentage of median BMI (ETD –8.24%; *P =* .049).

### 3.3 Insulin rescue use

In the liraglutide group, 15 participants were receiving basal insulin at baseline^21^ and 14 (25% of those who completed the trial) were doing so at week 52^31^ (*P =* .76) (Table 2). In the placebo group, the number of participants receiving basal insulin increased from 10 at baseline^21^ to 23 (43% who completed the trial) at week 52^31^ (*P =* .003) (Table 2). The mean insulin dose increased from 29.6 U at baseline in both the liraglutide and placebo groups to 31.0 U (liraglutide group) and 33.6 U (placebo group) at week 52.

**TABLE 2:** Basal insulin use at baseline and week 52

	Baseline	Week 52	*P* value
Liraglutide, n [%]	15 [22.7]	14 [25.0]	.76^*^
Placebo, n [%]	10 [14.7]	23 [43.0]	.003^*^

^*^ *P* value for the change in percentage of total participants using insulin from baseline to week 52. *P* values for the change in mean dose from baseline to week 52 were not calculated due to the small sample size. n, number of participants.

## 4 DISCUSSION

Results of our analysis demonstrated statistically significant reductions in relevant BMI and weight parameters with liraglutide compared with placebo from baseline to week 52, but not at week 26. Furthermore, comparing each of the three liraglutide doses individually with placebo, no significant dose‐dependent effects were observed using the primary imputation method. However, in the supporting analysis using the PMM with missing observations imputed from the placebo group (a more conservative method), there were significant differences in BMI and weight parameters observed between liraglutide 1.2 mg/day and placebo at week 52.

A smaller proportion of the participants in the liraglutide group compared with the placebo group who completed treatment at week 52 required insulin rescue (addition of basal insulin, alone or in combination with bolus insulin, to treat hyperglycaemia). This may have contributed to the greater effect on BMI and weight parameters with liraglutide compared with placebo, as insulin use is associated with weight gain.^11^ Therefore, the potential influence of weight gain with insulin therapy may have led to an overestimation of the efficacy estimate of liraglutide on weight parameters because fewer participants in the liraglutide group required insulin. However, these results likely offer a reasonable treatment effect with liraglutide that could be expected in the clinical setting, since insulin therapy is commonly used in this population.^32^ Therefore, regardless of the factors contributing to our findings, the results of this study provide evidence that treatment with liraglutide may result in a reduction in BMI and weight parameters in addition to improving glycaemic control in children and adolescents with T2D. These findings are consistent with the growing body of evidence suggesting that treatment with GLP‐1 RAs can result in significant BMI and weight reduction in children and adolescents with obesity, with or without T2D, as well as in adults with obesity, with or without T2D.^13^, ^14^, ^15^, ^16^, ^17^, ^18^, ^20^, ^21^, ^33^

It should be noted that the time course of changes in BMI and weight parameters observed in this analysis were generally similar to the BMI z‐score results reported in the ellipse trial primary paper, which showed a significant change in BMI z‐score from baseline with liraglutide compared with placebo at week 52, but no significant difference at week 26.^21^ One potential reason for the delayed treatment effect of liraglutide on BMI and weight parameters could be that only 55.6% of the participants in the liraglutide group reached the maximum dose of 1.8 mg/day.^21^ Although 1.8 mg/day is the maximum dose typically used for T2D in adults, and is currently approved by the FDA for children and adolescents, liraglutide doses of up to 3.0 mg/day have been found to be effective for BMI reduction in adolescents with obesity.^13^, ^16^, ^20^ We hypothesize that a higher degree of BMI and weight reduction may have been achieved in the ellipse trial if liraglutide had been increased to the maximum dose indicated for T2D (1.8 mg/day) in all participants in the liraglutide group. However, in some trials demonstrating weight reduction with liraglutide in children and adolescents, either no or only a small proportion of participants had T2D, which could limit the generalizability of these results to a population with T2D.^16^, ^20^ Additionally, as the participants' dose was limited by the study design and tolerability in each individual, and some tolerability issues may be experienced in a real‐world setting, it is unclear whether the hypothesized greater reduction in BMI at 1.8 mg/day would be observed in routine clinical practice.

Reductions in BMI and percent change in BMI with liraglutide were statistically significant compared with placebo; however, changes from baseline overall were modest given the patients' baseline severe obesity (mean BMI 33.9 kg/m^2^). Understanding the extent to which BMI must be reduced to positively impact body composition, metabolic health and cardiovascular disease risk is important to ensure that treatment interventions are appropriately designed and evaluated. Previous research has recommended to assess whether there is a continuous association between BMI and cardiovascular disease risk parameters, particularly if changes in risk parameters per BMI unit increase can be established.^34^ Others have suggested reductions of 0.2 in BMI z‐score might be clinically meaningful in children,^35^, ^36^, ^37^ although additional research has suggested that BMI and percent change in BMI are better alternatives to measuring adiposity.^23^ Overall, the impact of the reductions in BMI and weight parameters with liraglutide on broad health parameters among children and adolescents with severe obesity and T2D is unknown and requires additional assessment.

Weight reduction in adolescents with T2D is particularly important because previous literature has shown that weight loss, achieved through lifestyle interventions and/or bariatric surgery, can reduce insulin resistance and potentially contribute to the remission of T2D.^11^, ^38^, ^39^, ^40^, ^41^ Currently, recommendations for the management of T2D in children and adolescents state that patients with new onset T2D with an HbA_1c_ < 8.5% (69 mmol/mol) without ketosis or acidosis should be treated with metformin and lifestyle modification therapy as first‐line management.^42^, ^43^ However, evidence suggests that metformin is not associated with meaningful weight reduction,^8^, ^9^, ^44^, ^45^ and the Treatment Options for T2D for Adolescents and Youth (TODAY) study demonstrated that the median time to treatment failure (defined as HbA_1c_ ≥ 8.0% [64 mmol/mol] over 6 months or persistent metabolic decompensation^3^) was approximately 1 year with metformin monotherapy, metformin plus rosiglitazone, or metformin plus intensive lifestyle modification.^4^

International and US guidelines also recommend treatment with insulin if glycaemic goals are not met or if the initial HbA_1c_ is ≥8.5%, or in the presence of acidosis and/or diabetic ketoacidosis and/or other metabolic complications.^42^, ^43^ The most recent guidelines also mention to consider the addition of liraglutide to metformin, as well as initiating add‐on insulin or continuing insulin therapy, if glycaemic targets are not met. The addition of liraglutide can be considered if the child is over 10 years of age and there is no medical or family history of multiple endocrine neoplasia type 2 or medullary thyroid cancer.^43^

The recommendation to start insulin following metformin failure in children and adolescents contrasts with guidelines for adults with T2D, which recommend that other anti‐diabetes medications such as GLP‐1 RAs and sodium‐glucose co‐transporter‐2 inhibitors, many associated with weight loss or weight neutrality, can be started prior to insulin therapy, if medically appropriate.^10^, ^43^ These other pharmacotherapy options, which are indicated for adults but not for children and adolescents (with the exception of liraglutide), are recommended prior to insulin due to the weight gain associated with insulin, and subsequent increased insulin resistance.^10^, ^43^ Results from this current *post hoc* analysis suggest that liraglutide reduces BMI and weight parameters in children and adolescents with T2D and obesity. As ongoing relevant clinical trials are completed, resulting evidence may support further updates of paediatric clinical practice guidelines to be more closely aligned with adult guidelines.

This study has several limitations. First, this was a *post‐hoc* analysis, and the ellipse trial was not designed or powered to detect differences in BMI and weight parameters.^21^ Furthermore, the primary ellipse trial was not designed to assess the potential effect of insulin therapy on BMI and weight parameters in children and adolescents with T2D.^21^ Specifically, participants were not randomized to a liraglutide dose, nor were they necessarily titrated to the maximum liraglutide dose (1.8 mg/day).^21^ Liraglutide dose titrations were based on glycaemic effect and tolerability, which limited assessments of dose‐dependency effects.^21^ This limitation is readily evident with the 1.2 mg dose, where it appeared to provide the largest treatment effect. However, as this was based on data from just 12 participants, no firm conclusions about this can be made.

As with the primary results from the ellipse trial,^21^ the results may not be generalizable to all populations due to the somewhat limited diversity of the trial population. The majority of participants were White, although T2D among children and adolescents disproportionally affects Black/African American and Native American races^2^, ^46^; however, it is important to note that the ellipse study was multinational and not powered to determine effects by race/ethnicity. Furthermore, the impact of treatment unblinding at week 26 on BMI and weight parameters is unknown. Finally, a common limitation in trials with adolescents is the degree of missing data and suboptimal adherence to study protocols. However, two different statistical analyses (PMM with missing data imputed from the treatment group and PMM with missing data imputed from the placebo group) gave similar results, supporting the robustness of the data.

Further research is needed to evaluate the effect of liraglutide on decreasing or discontinuing insulin therapy, the effect of insulin rescue on BMI reduction in children and adolescents with T2D, and the effect of higher doses of liraglutide on BMI in children and adolescents with obesity and diabetes. Further randomized controlled trials in children and adolescents with T2D, with BMI and weight parameters as primary endpoints, could be useful to add to the growing body of research on the effect of liraglutide on weight outcomes.

In conclusion, in this *post‐hoc* analysis of the ellipse trial, liraglutide significantly reduced BMI (absolute and percent), %BMIp95, ΔBMIp95 and percentage of median BMI vs placebo at week 52, but not at week 26, with no significant dose‐dependency effects observed in children and adolescents with T2D. Further research is required to verify the suggested efficacy of liraglutide on BMI and weight parameters observed in this *post hoc*, secondary analysis.

## CONFLICT OF INTEREST

M.O.B. receives research support from Vivus Inc and serves as a site principal investigator.

E.M.B. serves as a site principal investigator for Novo Nordisk.

C.T.B. was funded by the National Institutes of Health's National Center for Advancing Translational Sciences, grants KL2TR002492 and UL1TR002494. The content is solely the responsibility of the authors and does not necessarily represent the official views of the National Institutes of Health's National Center for Advancing Translational Sciences. T.V.S.D., P.M.H. and C.K.R. are full‐time employees of Novo Nordisk; CKR and PMH also hold shares in Novo Nordisk.

KDR has no conflicts of interest to disclose.

ASK serves as an unpaid consultant for Novo Nordisk, Vivus and WW (formerly Weight Watchers), and receives drug/placebo from AstraZeneca for an NIDDK‐funded clinical trial.

## AUTHOR CONTRIBUTIONS

All authors contributed to the design of these *post‐hoc* analyses. Chethana K. Ramesh performed the statistical analyses. All authors contributed to the interpretation of the results. Megan O. Bensignor, Eric M. Bomberg, Carolyn T. Bramante, Kyle D. Rudser and Aaron S. Kelly wrote the first draft of the introduction and discussion. All authors reviewed each draft and approved the manuscript for submission.

## Supporting information

**Appendix S1.** Supporting information.
