# Supplementary material for: Effect of liraglutide treatment on body mass index and weight parameters in children and adolescents with type 2 diabetes: Post hoc analysis of the ellipse trial
Source: Pediatr Obes. 2021 Feb 25;16(8):e12778. doi: 10.1111/ijpo.12778 (PMC8277686; doi:10.1111/ijpo.12778)
Supplement: Supplementary file 1 — Appendix S1. Supporting information. [file IJPO-16-e12778-s001.pdf]

Supporting Information for:

Effect of liraglutide treatment on body mass index and weight parameters in children and adolescents with type 2 diabetes: *post hoc* analysis of the ellipse trial

Megan O Bensignor, MD[1,2]; Eric M Bomberg, MD[1,2]; Carolyn T Bramante, MD[2,3]; TVS Divyalasya, MD[4]; Paula M Hale, MD[5]; Chethana K Ramesh, MSc[4]; Kyle D Rudser, PhD[2,6]; Aaron S Kelly, PhD[1,2]

[1]Department of Pediatrics, University of Minnesota Medical School, Minneapolis, MN, USA

[2]Center for Pediatric Obesity Medicine, University of Minnesota Medical School, Minneapolis, MN, USA

[3]Division of General Internal Medicine, University of Minnesota Medical School, Minneapolis, MN, USA

[4]Novo Nordisk, Bangalore, India

[5]Novo Nordisk Inc., Plainsboro, NJ, USA

[6]Division of Biostatistics, University of Minnesota School of Public Health, Minneapolis, MN, USA

Corresponding author: Megan O Bensignor, Pediatric Endocrinology, Center for Pediatric Obesity Medicine, University of Minnesota, 717 Delaware St. SE, Office # 370 J, MN, USA. Phone: 612-626-2809; Fax: 612-301-6645; Email: moberle@umn.edu

Supplementary Table S1. Differences in mean change from baseline in BMI (absolute and percent change), %BMIp95, △BMIp95, percentage of median BMI, TMI and WC between placebo and liraglutide overall and by liraglutide dose at week 26 of the ellipse trial, using the PMM analysis with missing observations imputed from each randomized treatment group.

BMI/weight parameter (change from baseline)	n (liraglutide)	n (placebo)	Week 26† ETD (95% CI), *p* value
Absolute BMI (kg/m^2)			
Overall liraglutide – placebo	66	68	-0.35 (-0.99, 0.30), 0.29
Liraglutide 0.6 mg – placebo	19	68	-0.17 (-1.14, 0.80), 0.73
Liraglutide 1.2 mg – placebo	12	68	-0.81 (-1.92, 0.30), 0.15
Liraglutide 1.8 mg – placebo	35	68	-0.28 (-1.07, 0.51), 0.48
% change in BMI			
Overall liraglutide – placebo	66	68	-0.92 (-2.77, 0.92), 0.33
Liraglutide 0.6 mg – placebo	19	68	-0.30 (-3.14, 2.53), 0.83
Liraglutide 1.2 mg – placebo	12	68	-2.56 (-5.72, 0.61), 0.11
Liraglutide 1.8 mg – placebo	35	68	-0.69 (-2.93, 1.55), 0.55
%BMIp95			
Overall liraglutide – placebo	66	68	-1.74 (-4.98, 1.51), 0.29
Liraglutide 0.6 mg – placebo	19	68	-0.83 (-5.72, 4.05), 0.74
Liraglutide 1.2 mg – placebo	12	68	-4.10 (-9.73, 1.53), 0.15
Liraglutide 1.8 mg – placebo	35	68	-1.41 (-5.39, 2.57), 0.49
△BMIp95			
Overall liraglutide – placebo	66	68	-0.34 (-0.98, 0.30), 0.29
Liraglutide 0.6 mg – placebo	19	68	-0.17 (-1.13, 0.80), 0.73
Liraglutide 1.2 mg – placebo	12	68	-0.81 (-1.92, 0.30), 0.15
Liraglutide 1.8 mg – placebo	35	68	-0.28 (-1.06, 0.51), 0.49
% of median BMI			
Overall liraglutide – placebo	66	68	-2.01 (-5.75, 1.73), 0.29
Liraglutide 0.6 mg – placebo	19	68	-0.96 (-6.59, 4.68), 0.74
Liraglutide 1.2 mg – placebo	12	68	-4.73 (-11.23, 1.77), 0.15
Liraglutide 1.8 mg – placebo	35	68	-1.64 (-6.24, 2.96), 0.48
TMI			
Overall liraglutide – placebo	66	68	-0.14 (-0.55, 0.26), 0.48
Liraglutide 0.6 mg – placebo	19	68	-0.08 (-0.68, 0.53), 0.81
Liraglutide 1.2 mg – placebo	12	68	-0.40 (-1.10, 0.30), 0.26
Liraglutide 1.8 mg – placebo	35	68	-0.09 (-0.58, 0.40), 0.72
WC (cm)			
Overall liraglutide – placebo	66	68	-0.15 (-2.48, 2.19), 0.90
Liraglutide 0.6 mg – placebo	19	68	1.66 (-1.87, 5.20), 0.36
Liraglutide 1.2 mg – placebo	12	68	-1.01 (-5.05, 3.03), 0.62
Liraglutide 1.8 mg – placebo	35	68	-0.89 (-3.76, 1.98), 0.54

Data analyzed using a PMM of observed data with missing observations imputed within each randomized treatment group based on multiple (x10,000) imputations. Data were then analyzed with

ANCOVA model containing treatment (consisted of four groups: placebo and the three doses of liraglutide), sex and age group as fixed effects and baseline value as covariate. ETDs and CIs were combined using Rubin's formula. Participants categorized by dose taken for the longest time during the maintenance period, which consisted of the double-blind and open-label parts of the trial. †Change from baseline to week 26 in HbA_{1c} was the primary endpoint of the ellipse trial. %BMIp95, percentage of the 95th percentile for BMI; △BMIp95, difference in BMI from 95th percentile BMI; ANCOVA, analysis of covariance; BMI, body mass index; CI, confidence interval; ETD, estimated treatment difference from baseline; n, number of participants; PMM, pattern mixture model; TMI, tri-ponderal mass index; WC, waist circumference.

Supplementary Table S2. Differences in mean change from baseline in BMI (absolute and percent change), %BMIp95, △BMIp95, percentage of median BMI, TMI and WC between placebo and liraglutide overall and by liraglutide dose at Weeks 26 and 52 of the ellipse trial using the PMM analysis with missing observations imputed from the placebo group.

BMI/weight parameter (change from baseline)	n (liraglutide)	n (placebo)*	Week 26† ETD (95% CI), *p value*	Week 52, end of trial ETD (95% CI), *p* value
Absolute BMI (kg/m^2)				
Overall liraglutide – placebo	66	68	-0.31 (-0.93, 0.32), 0.33	**-0.92 (-1.72, -0.12), 0.024**
Liraglutide 0.6 mg – placebo	19	68	-0.15 (-1.09, 0.80), 0.76	-0.72 (-1.91, 0.48), 0.24
Liraglutide 1.2 mg – placebo	12	68	-0.80 (-1.90, 0.30), 0.16	**-1.41 (-2.82, 0.00), 0.050**
Liraglutide 1.8 mg – placebo	35	68	-0.23 (-0.99, 0.55), 0.57	-0.86 (-1.84, 0.11), 0.08
% change in BMI				
Overall liraglutide – placebo	66	68	-0.82 (-2.60, 0.97), 0.37	**-2.85 (-5.19, -0.52), 0.017**
Liraglutide 0.6 mg – placebo	19	68	-0.24 (-2.97, 2.50), 0.87	-2.29 (-5.91, 1.33), 0.22
Liraglutide 1.2 mg – placebo	12	68	-2.50 (-5.62, 0.63), 0.12	**-4.15 (-8.27, -0.03), 0.048**
Liraglutide 1.8 mg – placebo	35	68	-0.55 (-2.73, 1.63), 0.62	-2.72 (-5.52, 0.09), 0.06
%BMIp95				
Overall liraglutide – placebo	66	68	-1.55 (-4.72, 1.62), 0.34	**-4.65 (-8.69, -0.61), 0.024**
Liraglutide 0.6 mg – placebo	19	68	-0.72 (-5.49, 4.06), 0.77	-3.65 (-9.74, 2.44), 0.24
Liraglutide 1.2 mg – placebo	12	68	-4.03 (-9.61, 1.55), 0.16	**-7.15 (-14.28, -0.01), 0.050**
Liraglutide 1.8 mg – placebo	35	68	-1.14 (-5.04, 2.76), 0.57	-4.34 (-9.27, 0.59), 0.08
△BMIp95				
Overall liraglutide – placebo	66	68	-0.31 (-0.93, 0.32), 0.34	**-0.92 (-1.72, -0.12), 0.024**
Liraglutide 0.6 mg – placebo	19	68	-0.14 (-1.09, 0.80), 0.76	-0.72 (-1.93, 0.48), 0.24
Liraglutide 1.2 mg – placebo	12	68	-0.80 (-1.90, 0.30), 0.16	**-1.42 (-2.83, -0.01), 0.049**
Liraglutide 1.8 mg – placebo	35	68	-0.23 (-1.00, 0.55), 0.57	-0.86 (-1.84, 0.11), 0.08
% of median BMI				
Overall liraglutide – placebo	66	68	-1.79 (-5.45, 1.86), 0.34	**-5.36 (-10.01, -0.71), 0.024**
Liraglutide 0.6 mg – placebo	19	68	-0.82 (-6.33, 4.69), 0.77	-4.21 (-11.23, 2.80), 0.24
Liraglutide 1.2 mg – placebo	12	68	-4.66 (-11.10, 1.78), 0.16	**-8.24 (-16.46, -0.03), 0.049**
Liraglutide 1.8 mg – placebo	35	68	-1.33 (-5.83, 3.17), 0.56	-4.99 (-10.67, 0.68), 0.09
TMI				
Overall liraglutide – placebo	66	68	-0.13 (-0.53, 0.27), 0.52	-0.49 (-1.00, 0.02), 0.06
Liraglutide 0.6 mg – placebo	19	68	-0.07 (-0.67, 0.53), 0.82	-0.42 (-1.19, 0.35), 0.29
Liraglutide 1.2 mg – placebo	12	68	-0.40 (-1.09, 0.30), 0.26	-0.73 (-1.63, 0.17), 0.11
Liraglutide 1.8 mg – PBO	35	68	-0.07 (-0.56, 0.42), 0.79	-0.45 (-1.07, 0.17), 0.16
WC (cm)				
Overall liraglutide – placebo	66	68	-0.07 (-2.35, 2.20), 0.95	-1.57 (-4.40, 1.27), 0.28
Liraglutide 0.6 mg – placebo	19	68	1.57 (-1.87, 5.02), 0.37	0.47 (-3.83, 4.77), 0.83
Liraglutide 1.2 mg – placebo	12	68	-0.84 (-4.85, 3.17), 0.68	-3.71 (-8.72, 1.31), 0.15
Liraglutide 1.8 mg – placebo	35	68	-0.75 (-3.57, 2.07), 0.60	-1.98 (-5.44, 1.47), 0.26

Statistically significant results are in **bold**. Data analyzed using a PMM of observed data with missing observations imputed from the placebo group based on multiple (x10,000) imputations. Week 26 and 52 data were then analyzed with ANCOVA model containing treatment (consisted of four groups: placebo and the three doses of liraglutide), sex and age group as fixed effects and baseline value as covariate. ETDs and CIs were combined using Rubin's

formula. Participants categorized by dose taken for the longest time during the maintenance period, which consisted of the double-blind and open-label parts of the trial. *Due to the pattern mixture model, where missing observations were imputed, the n-numbers were the same for week 26 and 52. †Change from baseline to week 26 in HbA_{1c} was the primary endpoint of the ellipse trial. %BMIp95, percentage of the 95th percentile for BMI; △BMIp95, difference in BMI from 95th percentile BMI; ANCOVA, analysis of covariance; BMI, body mass index; CI, confidence interval; ETD, estimated treatment difference from baseline; n, number of participants; PMM, pattern mixture model; TMI, tri-ponderal mass index; WC, waist circumference.
